# *Retracted*: ‘A cross‐sectional qualitative analysis of the seeming split between nursing and midwifery professions in Ghana and the way forward’ by Michael Wombeogo

**DOI:** 10.1002/nop2.125

**Published:** 2018-01-29

**Authors:** Michael Wombeogo

**Affiliations:** ^1^ School of Allied Health Sciences Department of Public Health University for Development Studies Tamale N/R. Ghana

## Abstract

The above article in Nursing Open, published online on 29th January, 2018 in Wiley Online Library (http://onlinelibrary.wiley.com/), has been retracted by agreement between the journal Editor in Chief, Dr. Roger Watson and John Wiley & Sons Ltd. The retraction has been agreed due to unattributed overlap with other published material. The Editor‐in‐Chief carried out an investigation and concluded that there was no intention to deceive by the author.

**REFERENCE**

Wombeogo, M. (2018). A cross‐sectional qualitative analysis of the seeming split between nursing and midwifery professions in Ghana and the way forward. *Nursing Open*, *00*, 1–5. https://doi.org/10.1002/nop2.125

**Funding information**
The author declares that no funding has been received from any external or internal funders insofar as this manuscript is concerned.

### Abstract

**Aim**: To explore how Ghanaian nurses and midwives seem to split into autonomous professions and the perceived causes of such a split.

**Design**: Ten nurses and midwives from four main hospitals in the Tamale Metropolis, from the rank of senior nursing officer were involved in the study.

**Methods**: A qualitative cross-sectional in-depth interview was employed for the study among senior nurses and midwives in the hospitals in the Tamale Metropolis.

**Results**: Participants identified specificity of professional focus and split in training arrangement as the main causes. For instance, there are still differential gaps in the training and practice functions of nurses and midwives, different examinations and clear distinction in job descriptions of both professions. Power of association culture was considered a contributory factor. The study concludes that nursing and midwifery are two distinct but closely related professions that have similar characteristics and natural fusion of learning and work.

Ghana, midwifery, nursing, nursing and midwifery council, split, Tamale Metropolis

Retracted

### 1 | INTRODUCTION

Nurses and midwives are among the front-line service providers seeking to renew primary health care (PHC) and achieve universal health coverage in the health sector in Ghana. The relationship that existed between these professions is one admired and tagged as marriage. Development of nursing education in Ghana between 1957 and 1970 was characterized by dynamic change and growth. Following Ghana's independence, developments in nursing and midwifery education continued to be strongly influenced by external organizations and their designated experts (Abbey, 2005). Policies, such as the local training of nurses and midwives, provided impetus for nurses and midwives to further their education to assume senior positions in nursing/midwifery education and administration. Emphasis on training nurses/midwives to work in a hospital-based curative health system, which had been the legacy of colonialism, gradually shifted to abroad-based education that prepared health-care professionals to work in a variety of settings. Changes in nursing and midwifery education occurred in an economic climate that presented ongoing impediments, yet the vision of the first generation of Ghanaian health leaders facilitated the tremendous progress seen during this period.

Developments in nursing and midwifery have taking place in the context of growing demands for accessible and affordable high-quality care and the need for improved access to care and universal coverage (Nursing and Midwifery Council of Ghana (NMC), 2016). The World Health Organization ((WHO), 2006) has responded to this urgent need and several resolutions on strengthening nursing and midwifery services as have been passed by the World Health Assembly (2011), resolution WHA 64.7.

A more formidable way of enhancing the nurse and midwife's relation was to formulate a council to regulate them. The name of the council changed from Nurses and Midwives council of Ghana as provided for in the NRCD 117 of 1972 and LI 683 of 1971 in 2013 to Nursing and Midwifery Council following the enactment of the Health Professionals Regulatory Bodies Act 2013 (Act 857). The same Act mandates the council to secure in the public interest, the highest standards of training and practice of nursing and midwifery. The council carried a vision statement thus: to ensure the availability of trained nursing and midwifery professionals who would give competent, safe, prompt and efficient service for client delight (Nursing and Midwifery Council of Ghana (NMC), 2016).

Nursing is a profession in the healthcare sector focused on the care of individuals, families and communities so they may attain, maintain or recover optimal health and quality of life. Nurses may be differentiated from other healthcare providers by their approach to patient care, training and scope of practice.

According to International Council of Nurses (2016), nursing encompasses autonomous and collaborative care of individuals of all ages, families, groups and communities, sick or well and in all settings. Nursing includes the promotion of health, prevention of illness and the care of ill, disabled and dying people. Advocacy, promotion of a safe environment, research, participation in shaping health policy and in patient and health systems management and education are also key nursing roles.

Nurses practice in many specialities with differing levels of prescriber authority. Many nurses provide care in the ordering scope of physicians and this traditional role has shaped the public image of nurses as care providers. However, nurses are permitted by most jurisdictions to practice independently in a variety of settings depending on training level and area of focus. In the postwar period, nurse education has undergone a process of diversification towards advanced and specialized credentials and many of the traditional regulations and provider roles are changing.

Nurses develop a plan of care, working collaboratively with midwives, physicians, therapists, the patient, the patient's family and other team members, that focus on treating illness to improve quality of life. However, a midwife is a professional in midwifery, specializing in pregnancy, childbirth, postpartum, women's sexual and reproductive health (including annual gynaecological exams, family planning, menopausal care and others) and newborn care. They are also educated and trained to recognize the variations in the normal progress of labour and how to deal with deviations from normal to discern and what may intervene in high-risk situations, such as breech births, twin births and births where the baby is in a posterior position, using non-invasive techniques.

The International Confederation of Midwives (2015:1, http://internationalmidwives.org/who-we-are/policy-and-practice/icm-international-definition-of-the-midwife/) definition of midwifery states that:

> *A midwife is a person who has successfully completed a midwifery education program, that is duly recognized in the country where it is located and that is based on the ICM essential competencies for basic midwifery practice and the framework of the ICM global standards for midwifery education; who has acquired the requisite qualifications to be registered and or legally licensed to practice midwifery and use the title 'midwife': and who demonstrates competence in the practice of midwifery.*

In recent times, the Nursing and Midwifery Council (NMC) of Ghana, decided to see the two professions of nursing and midwifery as distinct and independent ones without recourse of one to the other in various respects. For instance, the NMC conducts different professional examinations for each of the professions, have separate schools for each of them and promotions and career advancement of for each are parallel to each other, but not the same, among others. The action of conducting separate licensure examinations for both professions seems to suggest that the NMC supports a split between the two professions. The NMC in recent times has put in place some measures to avoid duplication on both fields as was done in the past. In this case one is either a nurse or a midwife and not both.

#### 1.1 | Scope of practice for registered nurses and midwives

The Nursing and Midwifery Council of Ghana (NMC) understands that the existence of separate national registers for nurses and midwives may create confusion as to:

- Whether a person can work both as a nurse and a midwife and
- If the practice of nursing and midwifery is too distinct for overlap.

The NMC acknowledges that, while some areas of work are nursing specific, some are midwifery specific; there may also be common areas of practice to the two professions.

#### 1.2 | Common practice to nursing and midwifery professions

While nurses work with a diverse range of people, the focus of midwifery is specific. It is recognizable that certain elements of practice are common to nursing and midwifery. Examples of common areas relevant to each profession's scope of practice include: counselling, patient education, wound care and infection control, administration of medicines and care of gynaecological surgical patients. This may not be a comprehensive list of elements common to nursing and midwifery and that there is no standard approach. In addition, the Nursing and Midwifery Council of Ghana recognizes that some similarities exist in the range of issues that nurses and midwives may face. In acknowledgement of the ICN and ICM definitions of nurses and midwives, respectively, these similarities should be taken into account in the assessment of nurses and midwives and drawing a line of relationship between the two professions.

Retracted

Retracted

### 1.3 | Differences between nursing and midwifery

Nursing and midwifery are two distinct professions, although in practice many midwives are nurses. Nurses practice in many different clinical areas such as paediatrics, obstetrics, geriatrics, outpatient clinics and facilities, forensics, community health, thus a broader scope; whereas midwives specialize in providing care during pregnancy, birth and postpartum, for healthy women with low-risk pregnancies who are expecting healthy babies. A nurse may learn basic midwifery courses during training to be able to cover up in the absence of a midwife as part of his/her duties as a nurse and vice versa. Prior to the training of direct midwifery, one needed to be a nurse before becoming a midwife.

A midwife and a nurse do not specialize in the same thing. One is not higher than the other, just different specialties and different training for their jobs (Goudman, 2016). Although midwives are the go-to healthcare providers as Goudman (2016) puts it, for women who choose to deliver at home or in a freestanding childbirth centre, 95% of all the births they assist take place in hospitals. Midwives will handle all your prenatal care and will deliver your baby with as few medical interventions as possible. They often work in collaboration with a Medical Doctor (MD) and may call one in if complications arise; otherwise, they handle low-risk births from start to finish.

### 1.4 | Research questions

- What are the main differences between nursing and midwifery?
- What reasons have been adduced to prompt the decision for a supposed split between nursing and midwifery?
- What are the effects of the split between nursing and midwifery on health service delivery?
- What is the way forward for both professions in health service delivery?

### 1.5 | Research objectives

- To find out the main differences between nursing and midwifery professions
- To find out the reasons behind nursing and midwifery seeming split
- To assess the effect of the seeming split on the two professions and health service delivery
- To evaluate the way forward for both professions and have an impact on health service delivery

## 2 | METHODOLOGY

Research methodology comprises the Research setting, Ethical consideration, Research design, Data collection procedure, Sampling technique, Target population, Sample size determination, Tools for data acquisition, selection criteria, Data analysis technique and Limitations.

### 2.1 | Study design

The specific design adopted in this study is basically a cross-sectional qualitative analysis of the effects of the seeming split between nursing and midwifery profession in Ghana. Qualitative data are generally difficult to measure and quantify; yet it can reveal valuable attitudes and perspectives that can hardly be accessed through a traditional quantitative approach. The exploratory character of qualitative research permits the gathering of specific research, very often through an intensive dialogue between the interviewer and the respondent (van Maeen, 2002).

### 2.2 | Sample size

Convenient sampling was used to select 10 key informants; five Registered Nurses and five Registered Midwives.

### 2.3 | Data collection tool

Data collection was done by interviewing key informants. Their in-depth knowledge and perception on the seeming split between the two professions was used as the main tool for obtaining data.

### 2.4 | Sampling procedure

Convenient sampling was used. The participants were selected at random and no specified procedure was followed as the key informants were selected based on convenience to the researchers.

## 3 | RESULTS/FINDINGS

According to the research conducted, five of the six informants asserted to the claim of a split existing in the circles of the nursing and midwifery professions. One informant reiterated her uncertainty as to the extent of the split. All the key informants mentioned that there were similarities and clear-cut differences between nursing and midwifery, the latter being a specialty.

A significant revelation from the informants showed the actions of the Nursing and Midwifery council to be in support of the split. Hierarchical and examinations setups are conducted differently for certifying general nurses and midwives. The NMC has made provisions to avoid duplication of personnel on both fronts, thus one cannot be a nurse and a midwife at the same time.

Another key finding was the restriction of midwives to maternal, labour and ante natal wards only. While all other wards were open to nurses.

Two informants stressed that the rising maternal and child mortalities prompted the decision to give more attention to pregnant women, thus the split. One of the informants who turned out to be a ward in-charge said the man management of the two disciplines have become easier and more effective. She also believed it has significantly reduced the maternal mortality in the country as a whole.

Interestingly, the six key informants all agreed the split generally has more pros than cons, resulting in a better delivery of competent healthcare service to clients. One lecturer who doubled as one of the key informants suggested individual regulatory bodies be established, thus the Nursing council should be separated from the Midwifery council since there is split in both professions.

### 4 | DISCUSSION

This section discusses issues arising from the findings of the study related to the set objectives:

A nurse is a caregiver, provides care to patients in a variety of settings. This includes physical, which range from total care (doing everything for someone) to helping a patient with illness prevention. In addition, nurses care holistically for a patient. Midwives are specialists in normal pregnancy and birth and their role is to look after the pregnant woman and her baby throughout the phase of antenatal care, during labour and birth and up to 28 days after the baby has been born.

Nurses work in hospitals and medical facilities and they also work at surgeries, residencies, clinics, nursing homes, voluntary organizations and occupational health services, hospices as well as in the pharmaceutical industry. Midwives are generally the key health professionals who support, guide and care for the mothers, babies and families through pregnancy months, at the time of birth and in the postnatal period. Many midwives handle their own case load of women and also work in the community.

The rate at which maternal and infant mortality was sky rocketing was alarming and so the need for more attention to pregnant women in labour. Community nurses and general nurses alike were trusted to help salvage the carnage with the growing demand of maternal health and the lack of specialized skill. Nurses were constantly trailing in managing maternal complications; hence, the need to institutionalize the midwifery specialty. A professional midwife made a claim that obstetric haemorrhage was one of the many underlining fatalities nurses specialized in managing, which significantly reduced the number of maternal deaths in the country. Significantly, the ratio of nurse to patient increased as more general nurses were assigned to medical wards and more midwives recruited to serve in maternal and labour wards. That line of job separation spelt out clearly the roles of midwives and nurses.

For the past 4 years NMC has set different questions for final year nursing and midwifery students. This has shown clearly that the curriculums for these two respective professions are different, hence, so are their jobs. But it is important to realize that despite NMC-stated job descriptions of these two professions, hospital setting and management policies can either merge them or further split them during the discharge of nursing and or midwifery duties. That is to say, a nurse could work in labour ward of the hospital to assist in delivering even though it has been stated clearly that midwives are best suited for the jobs.

The NMC has taken a step further from organizing different examination questions for students to answer and has also prescribed different uniforms for these professionals at their work places. Most nurses shared their sentiments that the uniform adjudication has not just created a split between them in the eyes of the NMC council but also in the eyes of the many patients who come into the ward for care every time. This is what an informant had to say about the split:

> *"The effects of splitting these two inter-related disciplines have led to easier and more effective management. It has become easier to manage general nurses and midwives as separate professions. The Ghana Registered Midwives council was formed primarily to raise the standard of the practice of midwifery in Ghana to the highest level by promoting and protecting the interest and welfare of its members. The Ghana registered Nurses association is performing the same function for general nurses to provide better health care delivery in Ghana."*

Given the immense roles nurses and midwives play, be it specialized or general functions, it is important to continually ensure their effective functioning. That is, policies and inventions should be innovative enough to suit these two areas. If done, it would go a long way of significantly meeting the sustainable developmental goals as a nation.

In addition, the true split will enlighten professionals and students on their scope of work and the limitations. Individual regulatory bodies should be established; thus, the Nursing council should be separated from Midwifery Council since there is split in both professions and as much conduct separate examinations for its candidates.

#### 4.1 | Recommendations

Based on the findings, the split has been termed a blessing in disguise. Ghana's Ministry of Health may consider the nursing and midwifery professions to have separate councils to manage each of the two professions that will effectively scale up nursing and midwifery care towards the achievement of sustainable developmental goal 3. This could reduce 70% of maternal mortality in the country.

Nurses and midwives are on the front line of health service delivery. In many countries, such as Ghana, they are either leaders or key actors in multiprofessional, interdisciplinary health teams. Therefore, the Ministry of Health in Ghana should consider a legislative instrument to formalize any split that will maintain professional cohesion and team building.

### 5 | CONCLUSIONS

The findings of this research are important in terms of providing insights that may lead towards a new direction in addressing the seeming split between nurses and midwives in Ghana's health delivery machinery. The analysis reported in this paper brings together results of qualitative studies where experiences and perceptions of nurses

Retracted and midwives regarding the effects of the split between the nursing and midwifery professions are considered. The results of the study seek to provide guidance and a template for the nursing and midwifery bodies in Ghana to develop a consensus that is designed to benefit both professions in whichever situation.

This study has shown that nursing and midwifery professions are two distinct ones propelled primarily by the rise in maternal mortality in recent times. Thus, the need for intervention by giving more attention to pregnant women by training specialized people to take care of them. Hence, the need for a more focused training for midwives to play a centre stage in the fight for maternal and child survival.

### ACKNOWLEDGEMENTS

The author of this article is grateful to the respondents at the various health institutions and the nurses and midwives in the Tamale Metropolis in the northern region of Ghana for their candid participation and willingness to participate in this study. In addition, the author is grateful to the authors from whose work meaningful information has been tapped to enrich the literature review. All those who in diverse ways contributed by way of suggestions on one thing or the other are duly acknowledged. Above all, the authors are grateful to God for His mercies and sustenance at all times.

### CONFLICT OF INTEREST

The author declares that there is no conflict of interest in any form so far as the preparation and finalisation of this manuscript is concerned.

### AUTHOR'S CONTRIBUTION

Dr. Michael Wombeogo carried out data gathering with assistance on the field, did compilation, editing and finalization of research article for submission.

### ORCID

*Michael Wombeogo* http://orcid.org/0000-0003-3381-8231
